# Evaluating the Diagnostic Utility of 16S Oxford Nanopore Technology Sequencing in Patients With Central Nervous System Infections and Its Usefulness in Antimicrobial Stewardship

**DOI:** 10.1093/infdis/jiaf280

**Published:** 2025-05-26

**Authors:** Do Van Dong, Le Thi Kieu Linh, Nguyen Thi Tuyet Nga, Nghiem Xuan Hoan, Nguyen Thi Khanh Linh, Tran Thi Thanh Huyen, Hoang Xuan Quang, Tran Thi Lien, Van Dinh Trang, Vu Viet Sang, Peter G Kremsner, Le Huu Song, Dennis Nurjadi, Thirumalaisamy P Velavan

**Affiliations:** Institute of Tropical Medicine, Universitätsklinikum Tübingen, Tübingen, Germany; Institutue of Clinical Infectious Diseases, 108 Military Central Hospital, Hanoi, Vietnam; Vietnamese–German Center for Medical Research, Hanoi, Vietnam; Institute of Tropical Medicine, Universitätsklinikum Tübingen, Tübingen, Germany; Vietnamese–German Center for Medical Research, Hanoi, Vietnam; Institute of Tropical Medicine, Universitätsklinikum Tübingen, Tübingen, Germany; Institutue of Clinical Infectious Diseases, 108 Military Central Hospital, Hanoi, Vietnam; Vietnamese–German Center for Medical Research, Hanoi, Vietnam; Institutue of Clinical Infectious Diseases, 108 Military Central Hospital, Hanoi, Vietnam; Vietnamese–German Center for Medical Research, Hanoi, Vietnam; Institutue of Clinical Infectious Diseases, 108 Military Central Hospital, Hanoi, Vietnam; Vietnamese–German Center for Medical Research, Hanoi, Vietnam; Department of Microbiology, 103 Military Hospital, Hanoi, Vietnam; Department of Infectious Diseases, Viet Tiep Friendship Hospital, Haiphong, Vietnam; Department of Infectious Diseases, Hai Phong University of Medicine and Pharmacy, Haiphong, Vietnam; Department of Microbiology and Molecular Biology, National Hospital for Tropical Diseases, Hanoi, Vietnam; Institutue of Clinical Infectious Diseases, 108 Military Central Hospital, Hanoi, Vietnam; Vietnamese–German Center for Medical Research, Hanoi, Vietnam; Institute of Tropical Medicine, Universitätsklinikum Tübingen, Tübingen, Germany; Centre de Recherches Médicales de Lambaréné, Gabon; Institutue of Clinical Infectious Diseases, 108 Military Central Hospital, Hanoi, Vietnam; Vietnamese–German Center for Medical Research, Hanoi, Vietnam; Institute of Medical Microbiology, University of Lübeck and University Hospital Schleswig-Holstein Campus Lübeck, Lübeck, Germany; German Center for Infection Research, partner site Hamburg-Lübeck-Borstel-Riems, Lübeck, Germany; Institute of Tropical Medicine, Universitätsklinikum Tübingen, Tübingen, Germany; Vietnamese–German Center for Medical Research, Hanoi, Vietnam; German Center for Infection Research (DZIF), Partner Site Tübingen, Tübingen, Germany; Faculty of Medicine, Duy Tan University, Danang, Vietnam

**Keywords:** central nervous system, meningitis/encephalitis, cerebrospinal fluid, Oxford Nanopore sequencing, antimicrobial stewardship

## Abstract

**Background:**

Central nervous system (CNS) infections pose a significant public health challenge in resource-limited settings. Traditional culture-based and targeted molecular diagnostic methods have limitations in sensitivity and speed. This study retrospectively analyzed the data and cerebrospinal fluid (CSF) samples from our previous study to assess the diagnostic efficacy of untargeted 16S Oxford Nanopore Technology (ONT) sequencing compared to conventional CSF culture methods, with the goal of improving diagnostic accuracy, reducing time to treatment, and enhancing patient outcomes.

**Methods:**

A total of 329 patients from 4 hospitals were enrolled in the study. CSF samples were collected and processed for both CSF culture and 16S ONT sequencing. DNA were extracted from CSF and amplified for 16S rRNA sequencing using the MinION platform. Descriptive analyses were conducted to assess pathogen detection rates and the potential impact of sequencing on antimicrobial stewardship.

**Results:**

Of the 329 samples, 40 (12%) were positive for bacterial or fungal pathogens. 16S ONT detected pathogens in 28 samples (9%), while CSF culture identified pathogens in 23 samples (7%). 16S ONT sequencing identified 17 pathogens not detected by CSF culture, including *Streptococcus suis* and *Acinetobacter baumannii*. Based on 16S ONT findings, 61% of patients were found to have received inappropriate empirical antibiotic therapy and could have benefited from improved antimicrobial management, including de-escalation in 11, escalation in 5, and adjustments in 2 cases.

**Conclusions:**

16S ONT sequencing showed higher sensitivity and diagnostic yield than CSF culture, providing clinical insights for managing CNS infections through targeted antibiotic use and enhanced antimicrobial stewardship in resource-limited settings.

## Teaser

16S Oxford Nanopore Technology sequencing outperforms conventional cerebrospinal fluid culture in detecting pathogens in central nervous system infections, offering faster, more accurate diagnostics and improving antimicrobial stewardship in resource-limited settings.

Infections of the central nervous system (CNS), such as meningitis, encephalitis, and meningoencephalitis, can lead to severe and potentially life-threatening illnesses [1]. Timely diagnosis and prompt treatment are crucial for reducing complications, preventing irreversible damage, and improving survival rates in patients with CNS infections [2, 3]. While cerebrospinal fluid (CSF) culture remains the gold standard for diagnosing bacterial meningitis, it is time consuming with turnaround time up to 2 days, which can delay early intervention and worsen patient outcomes, especially in cases where patients have been pretreated with antibiotics [4].

CSF Gram staining is a widely used rapid diagnostic method that proves valuable in culture-negative cases. However, its sensitivity varies depending on the pathogens detected, and it is significantly reduced in patients who have received antibiotics prior to sampling, which may also contribute to underdiagnosis of antibiotic-resistant pathogens [4]. Studies comparing bacterial cultures with polymerase chain reaction (PCR)–based methods have demonstrated that molecular techniques, such as PCR, can detect pathogens in 30%–50% of culture-negative CSF samples, offering a valuable alternative when traditional cultures fail [5]. However, our previous study assessing the use of a targeted FilmArray Meningitis/Encephalitis panel to improve the diagnosis of meningoencephalitis in Vietnam found only a marginal improvement in diagnostic value in terms of sensitivity, and this is likely due to the specific local epidemiology [6].

The introduction of next-generation sequencing, particularly Oxford Nanopore Technology (ONT), has transformed pathogen diagnostics by offering faster turnaround times, lower costs, and the ability to simultaneously detect a wide range of pathogens from a single sample while avoiding the bias of targeted molecular diagnostic panels [7]. The 16S ribosomal RNA (rRNA) gene, a highly conserved marker found in nearly all bacteria, is a key target for sequencing. Its broad conservation across bacterial species enables the identification of a wide variety of bacterial pathogens, including fastidious organisms that may be difficult to detect with conventional culture [8]. Previous studies have shown that 16S ONT sequencing offers superiority compared to conventional microbiological cultures, with results that are valuable for both diagnostics and antimicrobial stewardship [9–12]. Moreover, the rapid and user-friendly library preparation process of ONT sequencing allows for timely pathogen identification, which can expedite clinical decision-making and improve patient management [13].

In Vietnam, a low- and middle-income country with limited diagnostic resources, CNS infections are caused by a diverse range of pathogens, which complicates diagnostic accuracy and increases the risk of undiagnosed cases. Studies have indicated that up to 75% of these cases remain undiagnosed, contributing to significant morbidity, mortality, and long-term sequelae [14]. This diagnostic gap places a significant burden on healthcare systems, leading to prolonged hospital stays, higher treatment costs, and reduced workforce productivity, all of which contribute to a considerable socioeconomic burden on the community [15, 16].

This study aims to assess the diagnostic efficacy of untargeted 16S ONT sequencing compared to traditional CSF culture methods, using CSF samples with the goal to improve diagnostic accuracy, reduce time to optimal antibiotic treatment, and ultimately enhance patient outcomes, especially in resource-limited settings.

## MATERIALS AND METHODS

### Study Cohort

This retrospective, multicenter, hospital-based cohort study was conducted between 1 July 2022 and 30 April 2023 across 4 hospitals in Vietnam: 108 Military Central Hospital, the National Hospital for Tropical Diseases, 103 Military Hospital, and Viet Tiep Friendship Hospital. The study cohort was from a previously published study [6]. Patients were eligible for the study if they had clinical signs of suspected CNS infections, based on the World Health Organization case definition modified by Dubot-Pérès et al [17]. Inclusion criteria were hospitalized patients with suspected meningitis or encephalitis (based on CNS infection symptoms such as fever, headache, nausea/vomiting, neck stiffness, focal neurological symptoms, seizures, and altered mental status) who underwent a lumbar puncture [18]. Exclusion criteria were (*i*) patients with contraindications to lumbar puncture, such as an intracranial space-occupying lesion with mass effect, a mass in the posterior fossa, abnormal intracranial pressure, or a local skin infection at the lumbar puncture site; (*ii*) patients with an incomplete clinical history; and (*iii*) patients who did not consent to the study.

### Patient Classification and Outcome Evaluation

The level of consciousness was assessed using the Glasgow Coma Scale (GCS), with a GCS score of <14 indicating altered mental status [19]. Clinical outcomes were evaluated using the Glasgow Outcome Scale (GOS), ranging from 1 (death) to 5 (mild or no disability/recovery). A favorable outcome was defined as a GOS score of 5, while unfavorable outcomes included death, vegetative state, and varying degrees of disability (GOS score 1–4) [20]. CSF pleocytosis refers to an increased number of white blood cells (WBCs) in the CSF indicating inflammation or infection in the CNS. Two thresholds based on the corrected WBC count were used to define pleocytosis: (*i*) ≥5 cells/μL, a lower threshold commonly used in clinical practice, especially in adults, to detect even mild inflammation; and (*ii*) ≥10 cells/μL, a higher threshold that can be used to increase specificity to reduce the likelihood of false-positive results. In CSF analysis, the corrected WBC count refers to the adjustment of the measured WBC count to account for peripheral blood contamination often caused by traumatic lumbar puncture [21]. Abnormal CSF glucose and protein levels were defined for all patients as values of <2.8 mmol/L or >4.2 mmol/L and <0.10 g/L and >0.25 g/L, respectively.

### Specimen Collection and Laboratory Tests

CSF sampling procedures were standardized across all centers to ensure consistency and reliability. CSF cultures were performed using the BACTEC Plus Aerobic/F System (Becton-Dickinson, Franklin Lakes, New Jersey, USA). In the event of bacterial growth, colonies were identified using the VITEK matrix-assisted laser desorption/ionization–time of flight mass spectrometry system, an automated microbial identification platform. Antimicrobial susceptibility testing was conducted with the VITEK 2 Compact System (bioMérieux, Lyon, France) to determine resistance profiles. Five hundred microliters of CSF was stored at −80°C and transported to Germany for 16S ONT sequencing, with strict cold chain protocols maintained throughout the process.

### CSF Sample Preparation and Nucleic Acid Extraction

DNA was extracted from 200 µL of CSF from each patient sample using the Quick-DNA HMW MagBead Kit (Zymo Research, Irvine, California, USA) for high-molecular-weight DNA extraction. DNA quality and quantity were assessed using the Qubit 4 fluorometer and the Qubit dsDNA HS Assay Kit (Thermo Fisher Scientific, Waltham, Massachusetts).

### 16S rRNA Sequencing With Oxford Nanopore's MinION Device

For multiplex sequencing, the 16S rRNA gene was PCR-amplified from CSF DNA using the 16S Barcoding Kit 24 V14 (SQK-16S114.24) (Oxford Nanopore Technologies, Oxford, United Kingdom), following the manufacturer's instructions. The PCR was carried out for 35 cycles at an annealing temperature of 55°C, using 10 ng of genomic DNA per reaction, with a nuclease-free water as negative control. Amplicons were purified using AMPure XP beads and quantified using the Qubit 4 fluorometer with the Qubit dsDNA HS Assay Kit. Bar-coded amplicons were pooled in equimolar ratios and sequenced on the MinION Mk1B (R10.4.1 flowcell) sequencer for 6 hours, using super-accurate base calling by MinKNOW software (v.24.11.10). After sequencing, the flow cell was cleaned using the Flow Cell Wash Kit (EXP-WSH004; Oxford Nanopore Technologies). To evaluate the validation parameters of our 16S rRNA sequencing method, we used the ZymoBIOMICS Microbial Community DNA Standard (Zymo Research Europe, Freiburg, Germany) as a positive control, which served as both an extraction control and a reference for sequencing performance. The resulting taxonomic profiles were compared to the manufacturer's theoretical composition to evaluate the accuracy and taxonomic resolution of our analysis pipeline.

### Data Analysis

Descriptive analyses were performed using R version 4.4.0 software (http://www.r-project.org). Sequencing data generated by MinKNOW were analyzed on the Nanopore EPI2ME cloud platform. Low-quality reads (Q score <10) and reads outside the 1400–1700 bp length range were discarded. Each read was classified at the species level based on a 90% coverage and a 97% identity threshold. The species with the highest number of aligned reads was considered the causative pathogen, unless identified in the negative control, in which case it was excluded from further analysis.

## RESULTS

### Overview of the Study Cohort and Clinical Characteristics

A total of 329 patients aged ≥18 years with suspected CNS infections were recruited from 4 hospitals around Hanoi, Vietnam, based on presenting symptoms such as fever, headache, and altered mental status. The patients were distributed among the hospitals as follows: 136 from the National Hospital for Tropical Diseases, 52 from 108 Military Central Hospital, 101 from Viet Tiep Friendship Hospital, and 40 from 103 Military Hospital. The main clinical characteristics are summarized in Table 1. In this study, only samples from 329 patients were available for sequencing. The mean age of the cohort was 54 years, and 68% were male. Underlying conditions included hypertension (27%), diabetes (19%), and cardiac disease (6%). Common clinical features included fever, headache, neck stiffness, and altered mental status. WBC count, protein, and glucose levels in CSF were significantly associated with positive CSF culture and 16S ONT sequencing results (Table 1).

**Table 1.** Demographic and Clinical Characteristics of Patients With Central Nervous System Infections

Characteristic		CNS Infections (n = 329)	16S ONT Positive (n = 28)	16S ONT Negative (n = 301)	*P* Value	CSF Culture Positive (n = 23)	CSF Culture Negative (n = 306)	*P* Value
Demographics								
Age, y, mean ± SD		54 ± 19	52 ± 18	55 ± 19	.386	49 ± 18	55 ± 19	.174
Male sex		224 (68)	21 (75)	203 (67)	.543	17 (74)	207 (68)	.697
Underlying conditions								
Hypertension		89 (27)	5 (18)	84 (28)	.356	3 (13)	86 (28)	.185
Diabetes		62 (19)	0 (0)	62 (21)	**.016**	1 (4)	61 (20)	.093
Cardiac disease		19 (6)	2 (7)	17 (6)	.670	1 (4)	18 (6)	1
Alcoholism		17 (5)	1 (4)	16 (5)	1	2 (9)	15 (5)	.337
Chronic liver disease		18 (6)	0 (0)	18 (6)	.382	2 (9)	16 (5)	.364
Chronic lung disease		17 (5)	2 (7)	15 (5)	.646	1 (4)	16 (5)	1
Kidney disease		15 (5)	0 (0)	15 (5)	.626	0 (0)	15 (5)	.611
Immunosuppressive drugs		13 (4)	0 (0)	13 (4)	.613	0 (0)	13 (4)	.611
Cancer		13 (4)	0 (0)	13 (4)	.613	0 (0)	13 (4)	.611
HIV		11 (3)	0 (0)	11 (4)	.608	0 (0)	11 (4)	1
Risk factors								
Post-neurosurgery		29 (9)	5 (18)	24 (8)	.086	5 (22)	24 (8)	**.041**
Head trauma		31 (9)	3 (11)	28 (9)	.737	2 (9)	29 (9)	1
CSF shunt		5 (2)	0 (0)	5 (2)	1	0 (0)	5 (2)	1
Clinical features								
Fever (>37.5°C)		272 (83)	23 (82)	249 (83)	1	22 (96)	250 (82)	.147
Headache		220 (67)	18 (64)	202 (67)	.925	19 (83)	201 (66)	.152
Neck stiffness		207 (63)	19 (68)	188 (63)	.718	20 (87)	187 (61)	**.024**
Nausea/vomiting		98 (30)	12 (43)	86 (29)	.172	12 (52)	86 (28)	**.028**
Seizure		29 (9)	2 (7)	27 (9)	1	0 (0)	29 (9)	.243
Focal neurologic deficits		20 (6)	0 (0)	20 (7)	.396	0 (0)	20 (7)	.38
GCS score, mean ± SD		13 ± 2	12 ± 3	13 ± 2	**.004**	11 ± 3	13 ± 2	**.001**
Altered mental status		142 (43)	20 (71)	122 (41)	**.003**	19 (83)	123 (40)	**<.001**
At least 2 of 4 symptoms recorded (headache, fever, stiff neck, altered mental status)		272 (83)	24 (86)	248 (82)	.798	22 (96)	250 (82)	.147
GOS score								
1: Death		32 (10)	2 (7)	30 (10)	.076	3 (13)	29 (9)	**.004**
2: Vegetative state		29 (9)	5 (18)	24 (8)		7 (30)	22 (7)	
3: Severe disability		64 (19)	5 (18)	59 (20)		1 (4)	63 (21)	
4: Moderate disability		62 (19)	1 (4)	61 (20)		2 (9)	60 (20)	
5: Mild or no disability		143 (43)	15 (53)	127 (42)		10 (44)	132 (43)	
CSF parameters								
WBC count, cells/μL^a^	Mean ± SD	1030 ± 5910	3392 ± 5072	804 ± 5937	**.016**	9601 ± 19 724	371 ± 1687	**.035**
	Pleocytosis of 5 cells/μL	224 (69)	24 (86)	200 (68)	.080	23 (100)	201 (67)	**.002**
	Pleocytosis of 10 cells/μL	174 (54)	22 (79)	152 (52)	**.011**	22 (96)	152 (51)	**<.001**
Protein, g/L^b^	Mean ± SD	1.56 ± 2.79	3.85 ± 4.82	1.35 ± 2.43	**.013**	5.36 ± 4.92	1.27 ± 2.32	**.001**
	Abnormal protein	311 (96)	24 (89)	287 (96)	.100	21 (91)	290 (96)	.26
Glucose, mmol/L^b^	Mean ± SD	4.16 ± 2.97	2.61 ± 2.22	4.30 ± 2.99	**.001**	1.01 ± 1.66	4.38 ± 2.91	**<.001**
	Abnormal glucose	187 (57)	19 (73)	168 (57)	.159	21 (96)	166 (55)	**.001**

Data are presented as No. (%) unless otherwise indicated. Significant *P* values are highlighted in bold.

Abbreviations: CNS, central nervous system; CSF, cerebrospinal fluid; GCS, Glasgow Coma Scale; GOS, Glasgow Outcome Scale; HIV, human immunodeficiency virus; ONT, Oxford Nanopore Technology; SD, standard deviation; WBC, white blood cell.

^a^The CSF leukocyte count was determined in 323 patients; CSF specimens from 6 patients had too many leukocytes for an exact count to be performed (missing values, n = 6 for both 16S ONT negative and CSF culture negative).

^b^The protein and glucose levels in the CSF were measured in 325 patients. For 16S ONT sequencing, 4 CSF samples were not analyzed (missing values: n = 3 for 16S ONT negative, n = 1 for 16S ONT positive). For CSF culture, the missing values were as follows: protein (n = 4 for CSF culture negative) and glucose (n = 3 for CSF culture negative, n = 1 for CSF culture positive).

### Pathogens Detected by 16S ONT and CSF Culture

**Figure 1.**
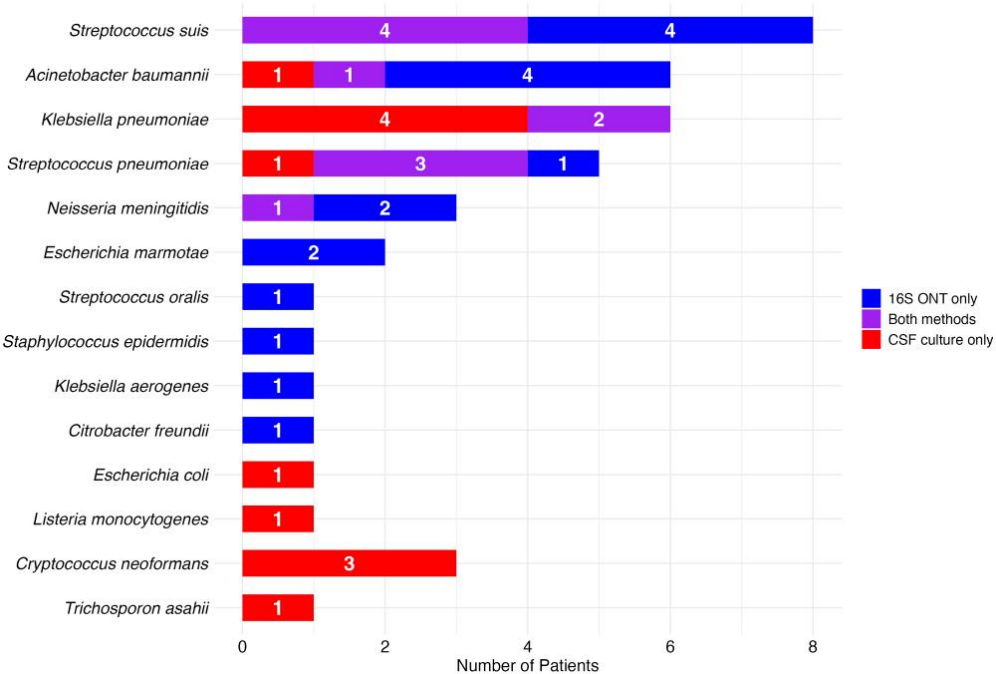
Bacterial pathogens detected using 16S ribosomal RNA Oxford Nanopore Technology (ONT) sequencing compared to cerebrospinal fluid (CSF) culture.

A total of 40 of 329 (12%) samples tested positive for bacterial or fungal pathogens, using either or both methods. Of these, 16S ONT sequencing identified pathogens in 28 (9%) samples, while CSF culture identified pathogens in 23 (7%) samples (Supplementary Figure 1). 16S ONT sequencing yielded a higher positivity rate than CSF culture in detecting *Streptococcus suis* (8 cases vs 4), *Acinetobacter baumannii* (5 vs 2), *Streptococcus pneumoniae* (4 vs 4), *Neisseria meningitidis* (3 vs 1), and *Klebsiella pneumoniae* (2 vs 6). 16S ONT sequencing detected 17 pathogens in 17 samples that were negative by CSF culture. This included *S suis* (n = 4), *A baumannii* (n = 4), *S pneumoniae* (n = 1), and *N meningitidis* (n = 2) as well as 5 pathogens detected exclusively by 16S ONT: *Escherichia marmotae* (n = 2), *Staphylococcus epidermidis* (n = 1), *Streptococcus oralis* (n = 1), *Klebsiella aerogenes* (n = 1), and *Citrobacter freundii* (n = 1) (Figure 1). The case descriptions for patients with positive 16S sequencing but negative CSF cultures, including relevant clinical information such as CSF parameters, documented diagnoses, and risk factors (eg, head trauma, recent neurosurgery, presence of shunts) supporting the likelihood of true infection, are listed in Supplementary Table 1.

**Figure 2.**
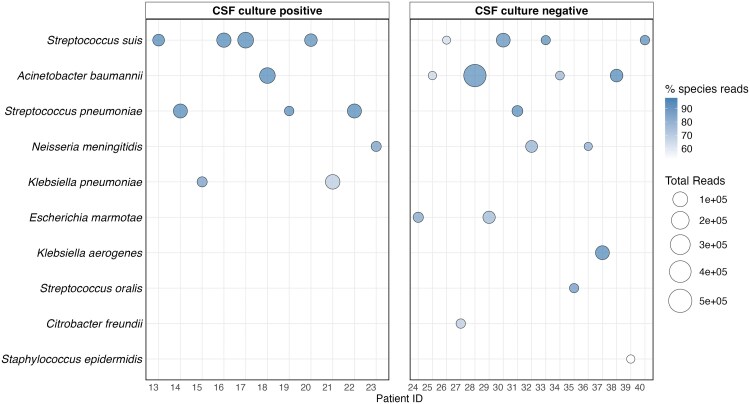
Causative pathogens detected with 16S ribosomal RNA sequencing using Oxford Nanopore Technology (n = 28). Size of the circles indicates the total number of sequencing reads. Abbreviation: CSF, cerebrospinal fluid.

In contrast, CSF culture detected 12 pathogens in 12 samples not identified by 16S ONT, including *A baumannii* (n = 1), *K pneumoniae* (n = 4), *S pneumoniae* (n = 1), *Cryptococcus neoformans* (n = 3), *Trichosporon asahii* (n = 1), *Listeria monocytogenes* (n = 1), and *Escherichia coli* (n = 1) (Figure 1). Nanopore-negative results were anticipated in 4 patients (IDs: 7, 9, 10, and 11), as *C neoformans* and *T asahii* are fungi not detectable by 16S rRNA sequencing (Supplementary Table 2). Moreover, the number of species-specific reads obtained from 16S rRNA ONT sequencing was higher in both CSF culture-positive and culture-negative specimens, further supporting our findings (Figure 2; Supplementary Table 3).

### Implications for Antimicrobial Stewardship

A retrospective analysis of clinical and laboratory data revealed that 61% (17/28) of patients with 16S ONT-positive results were initially prescribed unsuitable empirical antibiotics. If 16S ONT sequencing results had been available to guide therapy decisions alongside CSF culture, 18 patients would have benefited from this additional diagnostic method. This would have included de-escalation of antibiotic therapy for 11 patients, escalation for 5, and a change in antibiotics for 2 patients (Table 2).

**Table 2.** Characteristics of Patients Positive by 16S Ribosomal RNA Oxford Nanopore Technology Sequencing (n = 28)

Patient ID	Age/Sex	White Blood Cell Count	Glu	Pro	CSF Culture^a^	16S ONT^a^	Therapy Prior to Lumbar Puncture	Empirical Therapy	Treatment Adequacy	Appropriate Empirical Therapy	Possible Impact of 16S ONT on Treatment	Outcome^b^
13	51/M	1116	NA	1.56	*S suis*	*S suis*	No	CTX, CIP, MTP	CTX, CIP, MTP	No	Antibiotic de-escalation	5
14	41/M	10 460	0	1.65	*S pneumoniae*	*S pneumoniae*	Yes	CTX, LEV, MTP	CTX, LEV, MTP	No	Antibiotic de-escalation	5
15	37/M	199	4.33	6.09	*K pneumoniae*	*K pneumoniae*	Yes	MER, COL	MER, COL	Yes	No	2
16	61/F	599	1.08	6.1	*S suis*	*S suis*	Yes	CTX, DEX	CTX, DEX	Yes	No	5
17	44/F	9008	0.01	5.72	*S suis*	*S suis*	No	CTX, AMP, DEX	CTX, DEX	No	Antibiotic de-escalation	5
18	61/M	6903	0.01	22	*A baumannii*	*A baumannii*	Yes	MER, LIN, DEX	MER, COL	No	Antibiotic de-escalation	2
19	42/M	18 050	0.01	6.7	*S pneumoniae*	*S pneumoniae*	No	CTX, DEX	CTX, VAN, DEX	Yes	No	5
20	63/M	90	6	1.19	*S suis*	*S suis*	No	MER, AMK, MTP	MER, AMK, MTP	No	Antibiotic de-escalation	5
21	45/M	10 690	0	10.6	*K pneumoniae*	*K pneumoniae*	Yes	CTX, DEX	MER, COL, DEX	No	Antibiotic escalation	5
22	59/F	320	0.09	4.1	*S pneumoniae*	*S pneumoniae*	No	CTX, AMK, DEX	MER, AMK, DEX	No	Antibiotic de-escalation	1
23	20/M	8000	0	7.5	*N meningitidis*	*N meningitidis*	Yes	MER, CIP, DEX	CTX, CIP	No	Antibiotic de-escalation	5
24	50/M	240	3.34	0.92	Negative	*E marmotae*	Yes	CTX	MER	Yes	Antibiotic escalation	3
25	36/F	60	3.89	1.26	Negative	*A baumannii*	No	CTX, LEV	CTX, LEV	No	Antibiotic escalation	3
26	73/M	80	3.97	0.42	Negative	*S suis*	No	CFT	CFP, VAN	No	Antibiotic change	3
27	65/M	7	5.6	0.73	Negative	*C freundii*	Yes	MER, DEX	MER, DEX	Yes	No	1
28	74/M	3	4.4	0.37	Negative	*A baumannii*	No	TCC, LEV	TCC, LEV	No	Antibiotic escalation	2
29	35/F	2	3.6	0.37	Negative	*E marmotae*	No	No specific treatment	No specific treatment	NA	NA	5
30	86/F	2	4.2	0.31	Negative	*S suis*	No	CTX	CTX	Yes	No	5
31	74/M	5	7	0.67	Negative	*S pneumoniae*	No	MER, LEV, DEX	MER, LEV, DEX	No	Antibiotic de-escalation	3
32	84/F	1	4.4	0.92	Negative	*N meningitidis*	No	CPZ, LEV	CPZ, LEV	No	Antibiotic change	2
33	46/M	7641	4.42	2.01	Negative	*S suis*	Yes	MER, DEX	CTX, DEX	No	Antibiotic de-escalation	5
34	24/M	3779	0.06	2.97	Negative	*A baumannii*	Yes	MER, COL	MER, COL	Yes	No	4
35	58/M	13 993	3.78	11.7	Negative	*S oralis*	Yes	CTX, DEX	MER, VAN, DEX	Yes	No	5
36	31/M	641	3.3	1.95	Negative	*N meningitidis*	Yes	CTX, DEX	CTX, LIN, DEX	Yes	No	5
37	18/M	1308	0.23	NA	Negative	*K aerogenes*	Yes	MER, COL, DEX	MER, COL	Yes	No	2
38	68/M	38	3.55	0.6	Negative	*A baumannii*	Yes	MER, VAN, FLU	MER, VAN, FLU	No	Antibiotic de-escalation	3
39	37/M	1388	2.37	1.43	Negative	*S epidermidis*	No	CTX, ABD, DEX	ABD, DEX	No	Antibiotic escalation	5
40	59/M	355	0.7	4.1	Negative	*S suis*	Yes	CTX, TBM, DEX	CTX, TBM, DEX	No	Antibiotic de-escalation	5

Highlighted cells indicate patients who received antibiotics before sampling; 16S ONT sequencing could have potentially influenced treatment decisions in these cases if performed concurrently with CSF cultures.

Abbreviations: ABD, albendazole; AMK, amikacin; AMP, ampicillin; CFP, cefepime, CFT, cefoxitin; CIP, ciprofloxacin; COL, colistin; CPZ, cefoperazol; CSF, cerebrospinal fluid; CTX, ceftriaxone; DEX, dexamethasone, F, female; FLU, fluconazole; Glu, glucose; LEV, levofloxacin, LIN, linezolid; M, male; MER, meropenem; MTP, methylprednisolone; NA, not applicable; ND, not done; ONT, Oxford Nanopore Technology; Pro, protein; TBM, tobramycin; TCC, ticarcillin; VAN, vancomycin.

^a^ *A baumannii*, *Acinetobacter baumannii*; *C freundii*, *Citrobacter freundii*; *E marmotae*, *Escherichia marmotae*; *K aerogenes*, *Klebsiella aerogenes*; *K pneumoniae*, *Klebsiella pneumoniae*; *N meningitidis*, *Neisseria meningitidis*; *S epidermidis*, *Staphylococcus epidermidis*; *S oralis*, *Streptococcus oralis*; *S pneumoniae*, *Streptococcus pneumoniae*; *S suis*, *Streptococcus suis*.

^b^Outcome: 1 death; 2: vegetative state; 3: severe disability; 4: moderate disability; 5: mild or no disability.

## DISCUSSION

This multicenter study aimed to evaluate the diagnostic utility of 16S ONT sequencing for bacterial CNS infections across 4 hospitals in Hanoi, Vietnam, with a focus on improving diagnostic accuracy and antimicrobial stewardship in a region with a unique pathogen landscape. Our findings highlight the significant potential of 16S ONT sequencing to enhance pathogen detection, improve antibiotic therapy, and combat antimicrobial resistance (AMR), particularly in resource-limited settings.

### Diagnostic Performance of 16S ONT Sequencing

In this study, 16S ONT sequencing demonstrated a higher sensitivity when compared to traditional culture-based methods, as shown earlier [12, 22–24]. These findings indicate that 16S ONT sequencing can serve as a reliable complementary tool to CSF culture for the detection of bacterial pathogens in CNS infections, as documented from other studies [9, 11]. The sequencing method identified 28 pathogens in 28 CSF samples, with 23 pathogens also being detected by CSF culture. Notably, 16S ONT sequencing was able to detect 9 distinct pathogens in 17 culture-negative CSF samples, including key bacteria such as *S suis*, *A baumannii*, *N meningitidis, E marmotae*, *K pneumoniae*, *K aerogenes*, *S oralis*, *C freundii*, and *S epidermidis*. It is noteworthy that 9 of the 17 patients had received antibiotics prior to sampling, which may affect the viability of the causative pathogens and, thus, the overall sensitivity of culture-based methods. This highlights the superiority of sequencing-based methods, particularly in detecting fastidious or difficult-to-culture organisms. Another challenge for Vietnam is the atypical epidemiology of causative pathogens for community-acquired CNS. While *N meningitis* and *S pneumoniae* are common causes of meningitis worldwide, *S suis* is the leading bacterial pathogen of CNS infections in Vietnam and is associated with occupational exposure to pigs or consumption of raw pig products [25–27].

### Impact on Clinical Management

Vietnam is facing a major public health challenge as AMR continues to rise [28, 29]. This growing threat results from the inappropriate and excessive use of antibiotics, imposing a substantial financial burden on healthcare systems and society at large [30]. In clinical practice, when bacterial meningitis is suspected, empirical antibiotic therapy should be initiated as soon as possible. Given that microbiological results are typically unavailable at the time of initial treatment, clinicians must rely on local epidemiological data to guide their decisions. Once microbiological results are available, antimicrobial therapy should be reassessed and adjusted as necessary [31]. However, if microbiological results are negative, empirical broad-spectrum antibiotic therapy should be continued to address the possibility of antibiotic resistance [32].

The use of 16S ONT sequencing in our study sites would have a significant impact on the therapy decision and clinical management of the patients with CNS infections. In 18 of 28 cases, the sequencing results would have led to adjustments of the antibiotic therapy. Specifically, 11 patients would have benefited from antibiotic de-escalation, 5 would have required an escalation to broader-spectrum antibiotics, and 2 could have their treatment regimen tailored to match the identified pathogen. These findings underscore the value of rapid molecular diagnostics in guiding more precise and timely antimicrobial therapy in the sense of antimicrobial stewardship, which can reduce unnecessary antibiotic use and improve clinical outcomes. Moreover, by minimizing antibiotic overuse, the overall selection pressure on pathogens may be reduced, contributing to efforts to mitigate AMR in Vietnam, where the burden of infections with drug-resistant pathogens is high.

### False-Negative Results

Despite the overall good performance of 16S ONT sequencing, we observed some limitations in pathogen detection. For instance, *K pneumoniae* and *S pneumoniae* were identified more frequently by CSF culture than by ONT sequencing. Potential explanations for these discrepancies could be low bacterial load in CSF samples or challenges with the DNA extraction process. Improving the DNA yield by optimizing extraction protocols to better capture bacterial DNA from low-bacterial-load samples could help minimize false-negative results.

This study has a few limitations. First, the lack of a universally accepted gold standard for diagnosing bacterial meningitis complicates the assessment of diagnostic tools such as 16S ONT sequencing. Second, due to the retrospective nature of the study, not all patients underwent uniform testing. Notably, simultaneous serum glucose measurements were often unavailable, preventing calculation of the CSF/serum-glucose ratio, a key diagnostic marker, particularly in patients with diabetes or suspected hyperglycemia. Nonetheless, our study could show that 16S ONT sequencing could detect pathogens missed by conventional culture. Although 16S ONT sequencing demonstrated greater sensitivity than targeted molecular diagnostic panels, it should be viewed as a complementary tool rather than a replacement for culture-based diagnostics, serving to enhance overall pathogen detection.

In conclusion, 16S ONT sequencing is a promising tool for the diagnosis of bacterial CNS infections, offering significant advantages over traditional culture methods in terms of diagnostic yield, speed, and impact on clinical decision-making. By improving pathogen identification, guiding more accurate antibiotic therapy, and contributing to antimicrobial stewardship, 16S ONT sequencing has the potential to improve patient outcomes and combat AMR, particularly in resource-limited settings.

## Supplementary Material

jiaf280_Supplementary_Data

## Supplementary Data

Supplementary materials are available at *The Journal of Infectious Diseases* online (http://jid.oxfordjournals.org/). Supplementary materials consist of data provided by the author that are published to benefit the reader. The posted materials are not copyedited. The contents of all supplementary data are the sole responsibility of the authors. Questions or messages regarding errors should be addressed to the author.

## Notes

***Acknowledgments.*** We acknowledge support from the Open Access Publication Fund of the University of Tübingen.

***Author contributions.*** T. P. V. and D. N. designed the study. T. P. V. conceptualized the study, supervised the study, and contributed to the study materials and assays. D. V. D. performed the experimental procedures. D. V. D., N. T. T. N., and L. T. K. L. were involved in the analysis and validation of the results. D. V. D., N. X. H., T. T. T. H., N. T. K. L., H. X. Q., T. T. L., V. D. T., V. V. S., and L. H. S. recruited the patients and were involved in sampling procedures. D. V. D. wrote the first draft. T. P. V. reviewed and revised the first draft. P. G. K. and D. N. reviewed the manuscript. All authors have read and approved the manuscript.

***Ethical approval.*** This study adhered to ethical guidelines set by the Declaration of Helsinki to ensure participant safety and integrity. Written informed consent was obtained from all hospitalized patients and/or their relatives, and from parents for subjects <18 years of age. The study protocol was reviewed and approved by the institutional review board of the 108 Military Central Hospital, Hanoi, Vietnam (protocol number 108MCH/RES/MENTNGITIS-V-D3-25042017).

***Patient consent.*** Informed written consent was obtained from all hospitalized patients and/or their relatives after a detailed explanation of the study procedures.

***Disclaimer.*** The funder had no role in the study design, data collection and analysis, preparation of the manuscript, or decision to submit the manuscript for publication.

***Data availability.*** The 16S sequencing data have been deposited in the Sequence Read Archive under accession number PRJNA1261013. The authors confirm that the data supporting the findings of this study are available within the article and in its Supplementary Material.

***Financial support.*** This study was funded by German Academic Exchange Service (DAAD; Project ID: 5759243-PAN-ASEAN Coalition for Epidemic and Outbreak Preparedness [PACE-UP]).
