# Supplementary material for: Evaluating the Diagnostic Utility of 16S Oxford Nanopore Technology Sequencing in Patients With Central Nervous System Infections and Its Usefulness in Antimicrobial Stewardship
Source: J Infect Dis. 2025 May 26;232(2):e309–17. doi: 10.1093/infdis/jiaf280 (PMC12349954; doi:10.1093/infdis/jiaf280)
Supplement: jiaf280_Supplementary_Data [file jiaf280_supplementary_data.docx]

**Supplementary Table 1: Patients with positive 16S ONT but negative CSF culture results (n=17).**

| **Patient ID** | **Timepoint of sample collection** | **Risk factor** | **16S sequencing** | **Blood culture** | **Additional analysis** | **Summary of Clinical Diagnosis from Medical Records** |
| --- | --- | --- | --- | --- | --- | --- |
| 24 | During hospitalization | Head trauma, post-neurosurgery | *Escherichia marmotae* | Negative | NA | Bacterial meningitis |
| 25 | During hospitalization | Non-specific | *Acinetobacter baumannii* | NA | PCR (-ve) for *A.baumannii* | Meningitis of unknown cause |
| 26 | During hospitalization | Non-specific | *Streptococcus suis* | Negative | PCR (-ve) for *S. suis* | Meningitis of unknown cause |
| 27 | On admission | Non-specific | *Citrobacter freundii* | NA | NA | Septic shock, bacterial meningitis |
| 28 | During hospitalization | Non-specific | *Acinetobacter baumannii* | NA | PCR (-ve) for *A.baumannii* | Septic shock, bacterial pneumoniae |
| 29 | On admission | Non-specific | *Escherichia marmotae* | NA | NA | Migraine |
| 30 | On admission | Non-specific | *Streptococcus suis* | NA | PCR (-ve) for *S. suis* | Neurological Complications of Acute Rhinosinusitis |
| 31 | During hospitalization | Non-specific | *Streptococcus pneumoniae* | Negative | NA | Meningoencephalitis of unknown cause |
| 32 | During hospitalization | Non-specific | *Neisseria meningitidis* | Negative | NA | Septic shock, bacterial pneumoniae |
| 33 | On admission | Non-specific | *Streptococcus suis* | Negative | PCR (+ve) for *S. suis* | Bacterial meningitis |
| 34 | On admission | Head trauma, post-neurosurgery | *Acinetobacter baumannii* | Negative | PCR (+ve) for *A. baumannii* | Bacterial sepsis and meningitis |
| 35 | On admission | Non-specific | *Streptococcus oralis* | *S. oralis* | NA | Bacterial sepsis and meningitis due to *S. oralis* |
| 36 | On admission | Non-specific | *Neisseria meningitidis* | Negative | NA | Bacterial meningitis |
| 37 | On admission | Head trauma | *Klebsiella aerogenes* | Negative | NA | Bacterial meningitis |
| 38 | On admission | Post-neurosurgery | *Acinetobacter baumannii* | Negative | PCR (+ve) for *A. baumannii* | Bacterial sepsis and meningitis |
| 39 | On admission | Non-specific | *Staphylococcus epidermidis* | Negative | NA | Meningitis of unknown cause |
| 40 | On admission | Non-specific | *Streptococcus suis* | Negative | PCR (+ve) for *S. suis* | Bacterial meningitis |

**Abbreviation:** CSF, Cerebrospinal fluid; Patient ID; NA, Not Available; PCR, Polymerase Chain Reaction.

**Supplementary Table 2: Patients characteristics positive by CSF culture but negative 16S rRNA ONT (n=12).**

| **Patient ID** | **Age/Gender** | **Cell count** | **Glucose** | **Protein** | **CSF culture** | **16S ONT** | **Empirical therapy** | **Treatment Adequacy** | **Outcome** |
| --- | --- | --- | --- | --- | --- | --- | --- | --- | --- |
| 1 | 18/M | 3910 | 0.72 | 1.02 | *K. pneumoniae* | Negative | CFX | MER | 2 |
| 2 | 66/M | 1624 | 0.68 | 3.53 | *L. monocytogenes* | Negative | MER, VAN | MER, VAN, AMP | 2 |
| 3 | 31/F | 1960 | 0.2 | 2.13 | *S. pneumoniae* | Negative | CTX, DEX | MER | 5 |
| 4 | 23/M | 38731 | 0.1 | 6.34 | *A. baumannii* | Negative | MER, VAN, COL | MER, VAN, COL | 2 |
| 5 | 67/M | 91063 | 0.1 | 12.1 | *K. pneumoniae* | Negative | CTX, DEX | MER, AMK | 4 |
| 6 | 44/M | 9217 | 0.1 | 8.83 | *K. pneumoniae* | Negative | CTX, LEV, MET, DEX | MER, AMK | 4 |
| 7 | 45/M | 117 | 0.34 | 2.37 | *C. neoformans* | Negative | AMB, FLU, RHZ, S, MOX | AMB, FLU, RHZ, S, MOX | 2 |
| 8 | 44/M | 3024 | 0.32 | 2.41 | *K. pneumoniae* | Negative | MER, LIN | MER, DEX | 3 |
| 9 | 92/F | 135 | 3.65 | 0.44 | *C. neoformans* | Negative | CTX | AMB | 5 |
| 10 | 40/M | 445 | 2.4 | 1.71 | *C. neoformans* | Negative | MER, VAN | AMP, FLU | 1 |
| 11 | 78/F | 6 | 2.1 | 0.86 | *T. asahii* | Negative | MER, DEX | VCN, DEX | 1 |
| 12 | 62/M | 5157 | 0.02 | 8.3 | *E. coli* | Negative | MER, LIN, LEV | MER, LIN, LEV | 2 |

**Abbreviation:** Patient ID, Patient Identification; 16S ONT, 16S Oxford Nanopore Technology. F, Female; M, Male; ND, not done. AMB, Amphotericin B; AMK, Amikacin; AMP, Ampicillin; CFX, Cefotaxime; COL, Colistin; CTX, Ceftriaxone; DEX, Dexamethasone; FLU, Fluconazole; LEV, Levofloxacin; LIN, Linezolid; MER, Meropenem; MET, Metronidazole; MOX, Moxifloxacin; RHZ, Rifampicin-Isoniazid-Pyrazinamide; S, Streptomycin; VAN, Vancomycin; VCN, Voriconazole; Outcome: 1 death; 2: vegetative state; 3: severe disability; 4: moderate disability; 5: mild or no disability.

**Supplementary Table 3: 16S rRNA sequencing reads at genus and species level (n=28)**

| **Patient ID** | **Total reads** | **Results (Genus)** | **Genus reads** | **% genus reads** | **Results (Species)** | **Species reads** | **%** |
| --- | --- | --- | --- | --- | --- | --- | --- |
| 13 | 29166 | *Streptococcus* | 28586 | 98% | *Streptococcus suis* | 28566 | 98% |
| 14 | 76660 | *Streptococcus* | 74863 | 98% | *Streptococcus pneumoniae* | 74781 | 98% |
| 15 | 9131 | *Klebsiella* | 8391 | 92% | *Klebsiella pneumoniae* | 8237 | 90% |
| 16 | 84364 | *Streptococcus* | 82970 | 98% | *Streptococcus suis* | 82899 | 98% |
| 17 | 129709 | *Streptococcus* | 127728 | 98% | *Streptococcus suis* | 127629 | 98% |
| 18 | 124467 | *Acinetobacter* | 122356 | 98% | *Acinetobacter baumannii* | 122348 | 98% |
| 19 | 4300 | *Streptococcus* | 4181 | 97% | *Streptococcus pneumoniae* | 4180 | 97% |
| 20 | 44000 | *Streptococcus* | 42448 | 96% | *Streptococcus suis* | 42418 | 96% |
| 21 | 90235 | *Klebsiella* | 80579 | 89% | *Klebsiella pneumoniae* | 64529 | 72% |
| 22 | 77398 | *Streptococcus* | 75848 | 98% | *Streptococcus pneumoniae* | 75813 | 98% |
| 23 | 9225 | *Neisseria* | 8529 | 92% | *Neisseria meningitidis* | 8277 | 90% |
| 24 | 10556 | *Escherichia* | 9434 | 89% | *Escherichia marmotae* | 9183 | 87% |
| 25 | 194 | *Acinetobacter* | 134 | 69% | *Acinetobacter baumannii* | 130 | 67% |
| 26 | 34 | *Streptococcus* | 22 | 65% | *Streptococcus suis* | 22 | 65% |
| 27 | 3891 | *Citrobacter* | 3528 | 91% | *Citrobacter freundii* | 2802 | 72% |
| 28 | 441095 | *Acinetobacter* | 423520 | 96% | *Acinetobacter baumannii* | 423511 | 96% |
| 29 | 39166 | *Escherichia* | 32280 | 82% | *Escherichia marmotae* | 31004 | 79% |
| 30 | 72325 | *Streptococcus* | 68587 | 95% | *Streptococcus suis* | 68465 | 95% |
| 31 | 16515 | *Streptococcus* | 16037 | 97% | *Streptococcus pneumoniae* | 16026 | 97% |
| 32 | 31835 | *Neisseria* | 27105 | 85% | *Neisseria meningitidis* | 26201 | 82% |
| 33 | 3042 | *Streptococcus* | 2937 | 97% | *Streptococcus suis* | 2931 | 96% |
| 34 | 1138 | *Acinetobacter* | 922 | 81% | *Acinetobacter baumannii* | 915 | 80% |
| 35 | 3035 | *Streptococcus* | 2806 | 92% | *Streptococcus oralis* | 2799 | 92% |
| 36 | 97 | *Neisseria* | 83 | 86% | *Neisseria meningitidis* | 81 | 84% |
| 37 | 73999 | *Klebsiella* | 72522 | 98% | *Klebsiella aerogenes* | 72521 | 98% |
| 38 | 46062 | *Acinetobacter* | 44969 | 98% | *Acinetobacter baumannii* | 44966 | 98% |
| 39 | 122 | *Staphylococcus* | 67 | 55% | *Staphylococcus epidermidis* | 63 | 52% |
| 40 | 4321 | *Streptococcus* | 4164 | 96% | *Streptococcus suis* | 4157 | 96% |

**Abbreviation:** Patient ID, Patient Identification.

**Supplementary Figure 1:** Pathogens detected by CSF culture and 16S rRNA sequencing.


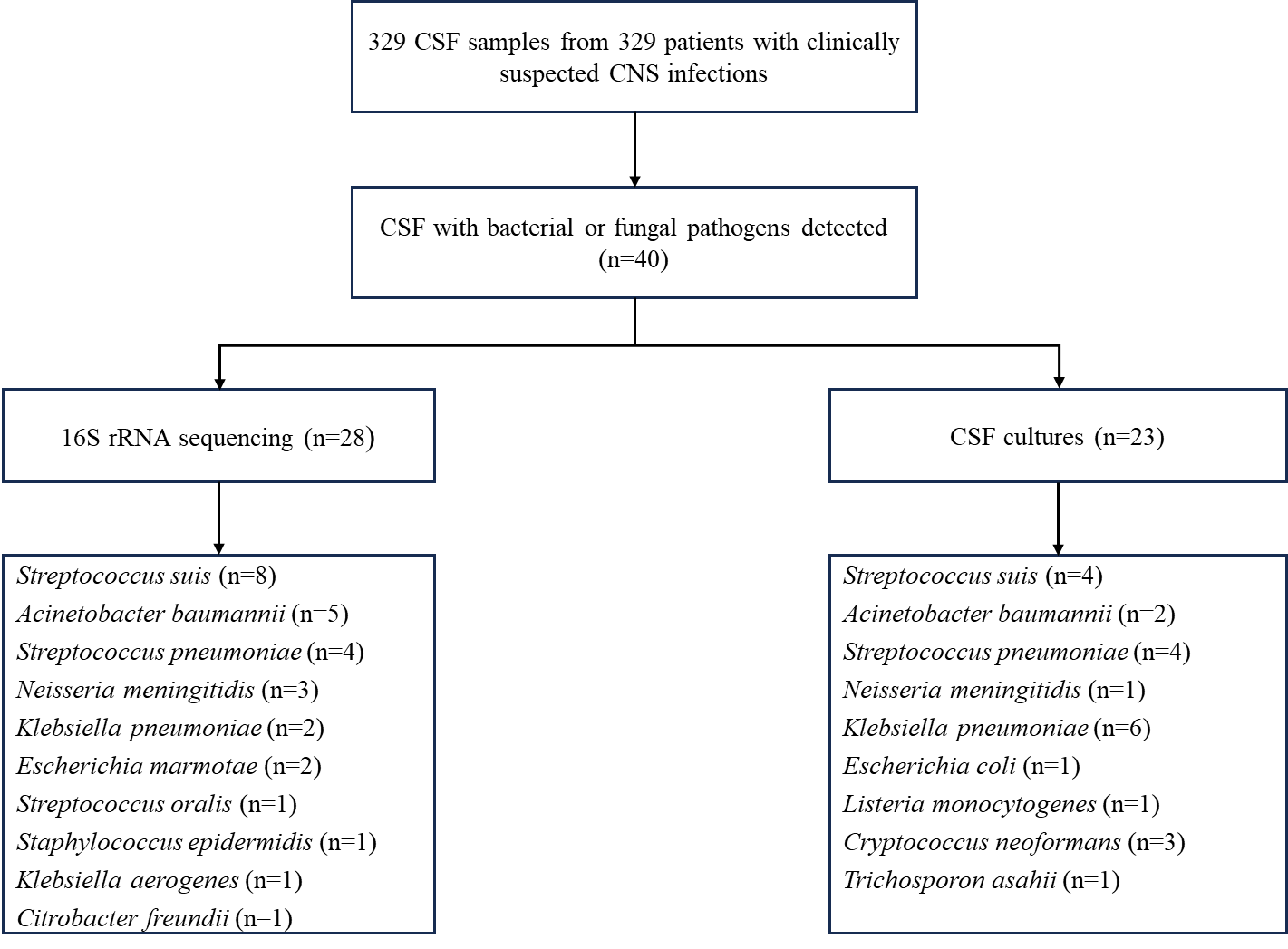


**Note**: 16S ONT sequencing yielded higher positivity rate than CSF culture in detecting *Streptococcus* *suis* (8 cases vs. 4), *Acinetobacter* *baumannii* (5 vs. 2), *Streptococcus pneumoniae* (4 vs. 4), and *Neisseria* *meningitidis* (3 vs. 1) and *Klebsiella* *pneumoniae*  (2 vs. 6). 16S ONT sequencing detected 17 pathogens in 17 samples which were negative by CSF culture. This included *S. suis (n=4)*, *A. baumannii (n=4)*, *S. pneumoniae (n=1)*, *N. meningitidis (n=2)* as well as five pathogens detected exclusively by 16S ONT: *Escherichia marmotae* (n=2), *Staphylococcus epidermidis* (n=1), *Streptococcus oralis* (n=1), *Klebsiella* aerogenes (n=1) and and *Citrobacter freundii* (n=1). In contrast, CSF culture detected 12 pathogens in 12 samples not identified by 16S ONT, including *A. baumannii* (n=1), *K. pneumoniae* (n=4), *S. pneumoniae* (n=1), *Cryptococcus neoformans* (n=3), *Trichosporon* *asahii* (n=1), *Listeria monocytogenes* (n=1), and *Escherichia* *coli* (n=1). Nanopore-negative results were anticipated in four patients as *C. neoformans* and *T. asahii* are fungi not detectable by 16S rRNA sequencing.
